# Editorial

**Published:** 1875-10

**Authors:** 


### Editorial.

### CELLULOID.

Yes, celluloid is gaining favor with the profession very rapidly; and it is proving much better than any ventured to anticipate, one ye ır ago. Many are now using it to the exclusion of all other cheap bases for artificial teeth. As now furnished, it is doubtless equal to, if not superior to rubber. It is more pleasantly and easily manipulated. The objection that seemed formerly to exist, viz: warping, change of color, and deterioration, have been overcome. There seems to be little doubt but that it will, in a short time, wholly supersede rubber for dental purposes. To those who have not yet used it we unhesitatingly say, give it a trial.

### THE LIST OF SOCIETIES.

It is desirable to keep our list of dental societies as accurate and complete as possible. Frequently, complaints come to us that the record is not at all correct, and this will necessarily be the case unless due notice is given of the changes that are made. It is very easy for the secretaries of the different societies to give us notice of the changes in their respective societies, as soon as they take place, and they will appear in the next issue of the REGISTER. If we are at the expense and labor of keeping up this list, it is certainly not asking too much that the officers send us correct statistics, so let us have them accurately and promptly.

### EASTERN INDIANA DENTAL ASSOCIATION.

The fifth annual meeting of the Eastern Indiana Dental Association will be held at Richmond, Ind., Tuesday and Wednesday, October 26th and 27th, 1875, commencing at 9 a. m., in Y. M. C. A. Rooms.

DISCUSSIONS.

1. Dental Literature.
   A. Code of Ethics.
2. Treatment of Diseased Gums.
3. Treatment of sensitive dentine and pulp exposures.
4. Filling Teeth.—
   A. Gold building vs. Gold stuffing.
   B. Amalgams, Hill's stopping, Oxy. chlo.
5. Instruments and Appliances.
6. Celluloid as a base for Artificial Teeth.

Miscellaneous business after each subject.

Clinics 8 A. M. to 12 M. on Wednesday.

Election of officers at 3:30 p. m. Wednesday.

Members are requested to have Essays or preparation for oral discussion on the different subjects.

Dentists not members and physicians are invited to attend our sessions. Prominent dentists have been invited and are expected to be with us.

EXECUTIVE COMMITTEE.

W. F. Shelley, New Castle; Milton H. Chappell, Kingstown; C. S. Wilson, Cambridge.

This meeting will be one of unusual interest, and the largest assembly since its organization.

### ASSOCIATION.

The Michigan Dental Association will hold its Annual Meeting at Cook's Hotel, Ann Arbor, commencing October 12th, 1875, at 12 o'clock M., and continue three days. The newly established Dental College being located at Ann Arbor, it has been deemed advisable by a large nunber of the profession to change the place of meeting from Grand Rapids to that City. It is hoped that all members of the profession will be present, as subjects of great interest will be brought up for consideration. All are cordially invited.

The following are the Committees appointed to report on subjects mentioned.

Anatomy,—J. A. Watling, W. L. Andrews, W. P. Morgan.

Physiology,—G. L. Field, C. E. Corbin, J. H. Woolley.

Chemistry,—C. S. Case, W. R. Cutler, J. W. Stom.

Hygiene,—D. C. Hawxhust, E. Hunter, D. W. Smith.

Pathology,—G. R. Thomas, H. H. Jackson, R. S. Bancroft.

Therapeutics,—J. C. Parker, J. A. Robinson, E. P. Cummings.

Surgery,—B. T. Spelman, E. G. Douglass, A. T. Metcalf.

Education,—W. H. Jackson, J. Lathrop, J. W. Finch.

Thomas R. Perry, President, W. D. Tremper, Secretary.

### IN MEMORIA.—DR. AARON BLAKE.

At a special meeting of the St. Louis Dental Society held on Tuesday Evening, August 24th, at the residence of Dr. Eames, the following preamble and resolutions of respect to the memory of Dr. Aaron Blake who died at his residence in St Louis, Aug. 23d, Aged 65, were unanimously adopted.

*Whereas*, It has pleased Our Heavenly Father to take from our midst, our beloved professional brother Aaron Blake who, by his integrity and arduous endeavours to accomplish his labors honestly and faithfully had endeared himself to his fellow members of this Society, and gained the universal respect, esteem, and regard of his professional brethren, therefore.

*Resolved*, That the St. Louis Dental Society deeply deploring his loss will revere his memory, and endeavor to follow his worthy example.

*Resolved*, That we deeply sympathize with the widow of the deceased in this hour of her great affliction and that we will manifest our affection and appreciation of his virtues by attending his funeral in a body.

*Resolved*, That a copy of these proceedings be spread on the minutes of this society, and a copy of the resolutions be forwarded to the widow. Also that a copy be sent to the Dental Journals for publication.

H. H. Keith, Sec.,

